# The Chinese HCT survey: a non-manipulated haploidentical transplantation procedure makes a novel contribution to data sharing within the regional and global transplant registries and to worldwide knowledge

**DOI:** 10.1038/s41409-021-01220-1

**Published:** 2021-01-29

**Authors:** Dietger Niederwieser

**Affiliations:** 1 University of Leipzig, Leipzig, Germany; 2 Lithuanian University of Health Sciences, Kaunas, Lithuania; 3 Aichi Medical University, Nagakute, Japan

**Keywords:** Medical research, Translational research

The article by Xu et al. [1] reports on 12,323 hematopoietic cell transplantations (HCT) by the Chinese Blood and Marrow Transplantation Registry Group (CBMTRG) in a single year (2019) contributing to a total of 58,914 HCT since 2008. Distinguishing features are the predominance of allogeneic (78%) over autologous HCT, the high frequency of haploidentical HCT (60%), the frequent use of bone marrow in combination with peripheral blood stem cells, and an increase in activity of 29% over the previous year. This is a unique experience worldwide.

The activity described by the CBMTRG is timely, impressive, and praiseworthy. After the first publication describing **21,884** HCT from 76 centers over 8 years (2008–2016) [2], the total numbers of HCT have doubled in just 3 years, and the reporting transplant centers increased threefold. The Chinese activity with 12,323 HCT annually is now comparable to that of the entire Asian Pacific region (SEAR/WPR) in 2015 without China (13,032 HCT) [3]. Within the global survey of the Worldwide Network for Blood and Marrow Transplantation (WBMT), China is approaching in 2016 the number of teams in North America (*n* = 215) and the median HCT/team (82.7 in China versus 93.7 in North America). Considering its population size (**1,440** million), China has achieved a remarkable transplant rate (TR; HCT/10 million population) of 86 (Fig. 1) in comparison to 54 in the whole SEAR/WPR and a Team Density (TD; teams/10 million population) of 1.03 [4]. Most importantly, the registry collects the activity every 6 months and is compatible with the Asian Pacific Blood and Marrow Transplantation (APBMT) registry, which contributes to the worldwide survey of the WBMT.

**Fig. 1:**
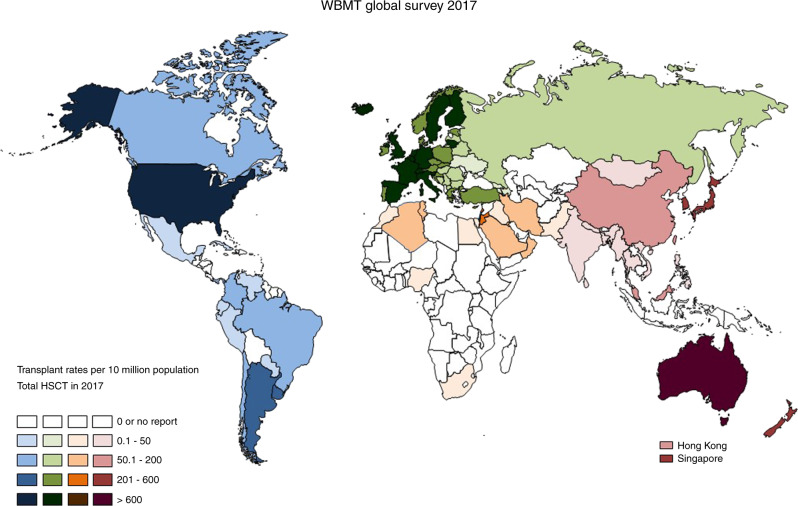
Transplant rates according to countries. Please note that after establishing the Chinese Blood and Marrow Transplantation Registry Group China advanced from the lowest category in 2016 to the intermediate category (50.1-200 HCT/10 million population) in 2017.

## Why is it important to maintain an accurate national, international, and global activity survey?

The whole art of medicine is supported by four important doctrines: observe, record, tabulate, and communicate [Sir William Osler (1849–1919)]. By observing, recording, and finally communicating medical experience we increase the knowledge in the medical field in general and particularly in this highly specialized treatment. HCT is the only curative treatment available for many diseases and is considered a routine although complex procedure. Driven by the necessity to adapt to local resources and availability of donors, a constant evolution is in progress in the different world regions. This local specialization threatens to diminish treatment and technology diffusion worldwide. It is therefore of crucial importance to share information on indications, on specific technologies and trends in HCT to prepare the necessary infrastructure and to avoid errors in planning. To ensure equity, quality and financial risk protection, the guiding principles of the World Health Organization (WHO; www.who.org) declare the transplantation of organs, cells, and tissue to be a global task. The collection of activity is certainly one of the prime prerequisites to identify differences between countries of dissimilar background and income and to support diffusion.

Scientific societies have an essential role to play in exchanging and collecting information on HCT. The need for scientific societies led in the 1970s to the foundation of the Center for International Blood & Marrow Transplant Research (CIBMTR) in North America and the European Society for Blood and Marrow Transplantation (EBMT) in Europe. APBMT for the Asia Pacific region, the World Marrow Donor Association (WMDA) and the East Mediterranean Blood and Morrow Transplantation (EMBMT) followed. Finally, with the intention of promoting HCT globally, four scientific societies (CIBMTR, EBMT, APBMT, and WMDA) founded the umbrella organization WBMT in 2006 (www.wbmt.org). The WBMT now comprises more than 20 societies working on different aspects of HCT. As a nongovernmental organization in working relation with the WHO from the beginning, the WBMT has taken up the challenge of collecting and disseminating worldwide HCT.

The main tools available to the WBMT are the analysis of activities and trends worldwide, the creation of regional scientific societies, and in cooperation with WHO and local health authorities, the organization of workshops in different world regions. After the foundation of the Latin American Blood and Marrow Transplantation Group (LABMT) and the African Blood and Marrow Transplantation Group (AFBMT) an extensive, worldwide network was established. Starting in 2006, the WBMT undertook biannual publications of the frequencies of HCT worldwide through regional societies or, in location without a regional registry, directly from transplant centers [5–9]. Although overall coverage was high, gaps remained in extremely large countries like China. With the published Chinese activity, a big gap has been largely closed.

The organization of workshops in countries with low activity was an important tool to reach global reporting and increased diffusion using education, twinning, and telemedicine. The main target was to share the accumulated experience covering a range of past errors and successes, but taking into account the regional needs. The final aim was to improve access for all patients in need and to establish a global model for the application of medical products of human origin. Successful meetings cosponsored by the WHO were hosted in Vietnam, Brazil, South Africa and Saudi Arabia. The read out for such meetings were subsequent increases in activities and reporting. One of the workshops was held 2018 in Beijing (China) with the special intent to merge the different registries in the country. The article published in this issue is one of the fruits of this workshop.

## What data are collected in HCT registries?

According to their scope, we distinguish between surveys and outcome registries. Surveys are activity registries looking at HCT numbers in individual centers documenting donor type, donor matching, stem cell source, disease type, and stage. Since 1957, 1.5 million procedures have been reported worldwide and more than 82,000 HCT collected in 2016 from >1600 transplant centers [4]. Such registry data are important for physicians, health authorities, WHO and WHO member States to detect indications, trends, to provide transparency, avoid inequalities, and learn from each other’s. The annual European surveys are important examples [10].

Outcome registries are collecting instead patient-specific information usually by data managers who collates minimal essential longitudinal outcome data (MED A; TED) and detailed patient, donor, disease and method specific information (e.g., MED B). Outcome registries need adequate databases and quality controls. It is not surprising that outcome registries are usually missing in countries with restricted resources. Here, the WBMT is helping to establish outcome registries, as these will be an essential part of the transplant procedure for quality checking, for listing side effects and complications. Analysis of such outcome registries have influenced daily praxis work such as timing of HCT in CML [11] and selection of unrelated donors [12].

Having now established the national survey, the Chinese Registry is narrowing an essential gap in the completeness of global information. Because of its size (14.9% of worldwide HCT) the provided information will significantly increase the power of the global survey and boost the information on HCT in the Asia Pacific Area. The unique information on haploidentical HCT and multiple stem cell sources included in the Chinese registry reports a new successful method for treating patients without a matched donor that adds to the in vitro T-cell depletion and the post-cyclophosphamide techniques. Overall, the Chinese registry is a perfect model of national and international collaboration, exchange, and humanity. The outcome registry is the next step to target.
